# Stochastic Process Underlying Emergent Recognition of Visual Objects Hidden in Degraded Images

**DOI:** 10.1371/journal.pone.0115658

**Published:** 2014-12-26

**Authors:** Tsutomu Murata, Takashi Hamada, Tetsuya Shimokawa, Manabu Tanifuji, Toshio Yanagida

**Affiliations:** 1 Center for Information and Neural Networks, National Institute of Information and Communications Technology (NICT), and Osaka University, Suita, Osaka, Japan; 2 Advanced ICT Research Center, NICT, Kobe, Hyogo, Japan; 3 Graduate School of Frontier Biosciences, Osaka University, Suita, Osaka, Japan; 4 Laboratory for Integrative Neural Systems, RIKEN Brain Science Institute, Wako, Saitama, Japan; CSIC-Univ Miguel Hernandez, Spain

## Abstract

When a degraded two-tone image such as a “Mooney” image is seen for the first time, it is unrecognizable in the initial seconds. The recognition of such an image is facilitated by giving prior information on the object, which is known as top-down facilitation and has been intensively studied. Even in the absence of any prior information, however, we experience sudden perception of the emergence of a salient object after continued observation of the image, whose processes remain poorly understood. This emergent recognition is characterized by a comparatively long reaction time ranging from seconds to tens of seconds. In this study, to explore this time-consuming process of emergent recognition, we investigated the properties of the reaction times for recognition of degraded images of various objects. The results show that the time-consuming component of the reaction times follows a specific exponential function related to levels of image degradation and subject's capability. Because generally an exponential time is required for multiple stochastic events to co-occur, we constructed a descriptive mathematical model inspired by the neurophysiological idea of combination coding of visual objects. Our model assumed that the coincidence of stochastic events complement the information loss of a degraded image leading to the recognition of its hidden object, which could successfully explain the experimental results. Furthermore, to see whether the present results are specific to the task of emergent recognition, we also conducted a comparison experiment with the task of perceptual decision making of degraded images, which is well known to be modeled by the stochastic diffusion process. The results indicate that the exponential dependence on the level of image degradation is specific to emergent recognition. The present study suggests that emergent recognition is caused by the underlying stochastic process which is based on the coincidence of multiple stochastic events.

## Introduction

Visual object recognition requires a match to be established between an input image and an appropriate object representation stored in the high-level visual system [1], [2]. When an image is severely degraded, as in the cases of the “Dalmatian dog” [3] or Mooney images [4]–[8], it appears meaningless when seen for the first time because the bottom-up processing of the image, whose descriptions of objects are partly lost owing to degradation, does not provide sufficient information to determine an object representation to match. This deficit of information can be supplemented by top-down processing, in which higher-order cognitive processes of expectations or attentional controls based on knowledge about the object guide lower levels of processing such as interpolation or segmentation of the defective image [9], [10]. Many studies have shown that the top-down processing of object information, which is provided by previous viewing of the original undegraded image [5]–[8], [11]–[13], a visual context [14], or an instruction using a nonvisual context [15], [16], effectively facilitates recognition of an object hidden in a degraded image. However, even in the absence of any such information for top-down processing, with continued observation, hidden objects in degraded images can be recognized in an emergent manner, being frequently accompanied by a feeling similar to the “Aha!” [13] or Eureka experience [17]. This emergent recognition (ER) is characterized by a comparatively long reaction time (RT) ranging from seconds to tens of seconds, whereas detection or recognition of unambiguously depicted objects typically requires a RT of within a second or so as shown by many studies [18]–[21]. This long RT, which has seldom been investigated in quantitative studies, does not seem to be explained by simple combinations of bottom-up and top-down processes, suggesting that a separate neural mechanism is involved in the search for object representations that can match defective input patterns.

To explore this time-consuming process of ER, we measured the RTs for recognition of degraded images in this study. Subjects (*n* = 91) were asked to recognize degraded images of various objects. In total, 90 degraded images of objects in various categories were used, all of which were newly created through monochromatic binarization of color images to prevent recognition based on prior knowledge of the objects. Our results demonstrated that the time for recognition of degraded images follows a particular exponential function, which is determined by two parameters: subject's capability and task difficulty caused by image degradation. To explain this function, we developed a neurophysiology-inspired theoretical model of stochastic process. In this model, although some components of the object's representation are eliminated by image degradation, a stochastic process is employed to search for the missing components that complete the representation of the object. This process requires a certain amount of time, which explains the delay in RTs for recognition of degraded images. The results showed that our model successfully accounted for the function obtained in the experiment, suggesting that the stochastic search process underlies the ER.

To see whether the obtained results were specific to the ER task, we also conducted a comparison experiment in which the task of perceptual decision making that is known to involve a certain stochastic process was compared with the ER task. The result showed a remarkable difference in their RT distributions suggesting that the exponential increase of time according to task difficulty may be a characteristic specific to ER.

## Results

### Measurement of time for recognition of degraded images

**Figure 1:**
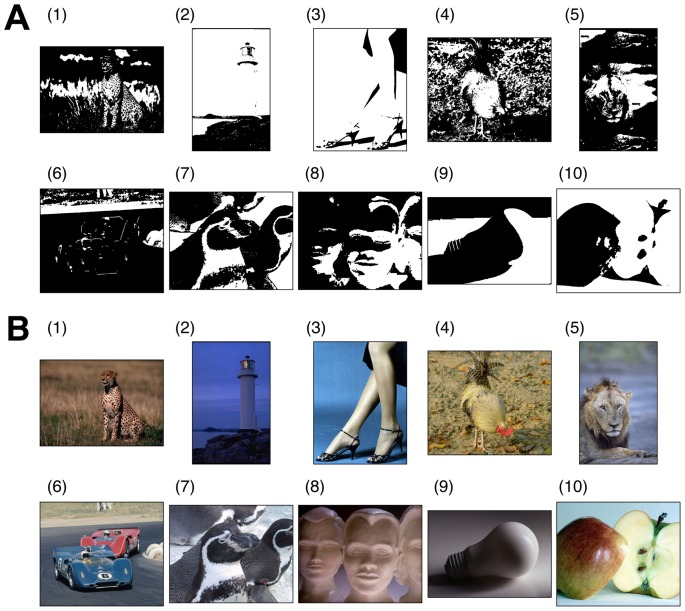
Examples of degraded images and their original color images used in the experiment. (A) Degraded images were created using monochromatic binarization of color images (see Materials and Methods). The examples represent various levels of difficulty. The numerical labels used in Figs. 1B, 2A, 2B, 5A, 5B, and 8 are consistent with those presented here. (B) The original images of the examples in Fig. 1A.

Ninety new degraded images were created using monochromatic binarization of color images, each of which contained a distinct object that could be easily described verbally. Examples of the degraded images and their original color images are shown in Figs. 1A and 1B, respectively. Subjects were instructed to press a key as quickly as possible when they recognized something meaningful in each degraded image presented on a monitor. The image on the monitor was turned off immediately when the key was pressed, and the RT was recorded. Subjects were then required to report verbally what they had recognized to the experimenter. Although a degraded image may have multiple interpretations, only the object shown in the original image was accepted as correct. So that trials were not interminable, the presentation time of each image was limited to 30 s, after which the image was turned off without revealing the correct answer, and the next trial was initiated. To estimate delay in RT for recognition of each degraded image, separate sessions were conducted for measuring RTs for recognition of degraded images and their original color images. We subtracted RT values for the original images from those for their corresponding degraded images, assuming that this subtraction eliminates the time taken for the common processes. We refer to this time difference as the “search time” required for recognition of degraded images, based on the idea that this delay reflects the time-consuming search process for an object representation that matches the degraded image.

**Figure 2:**
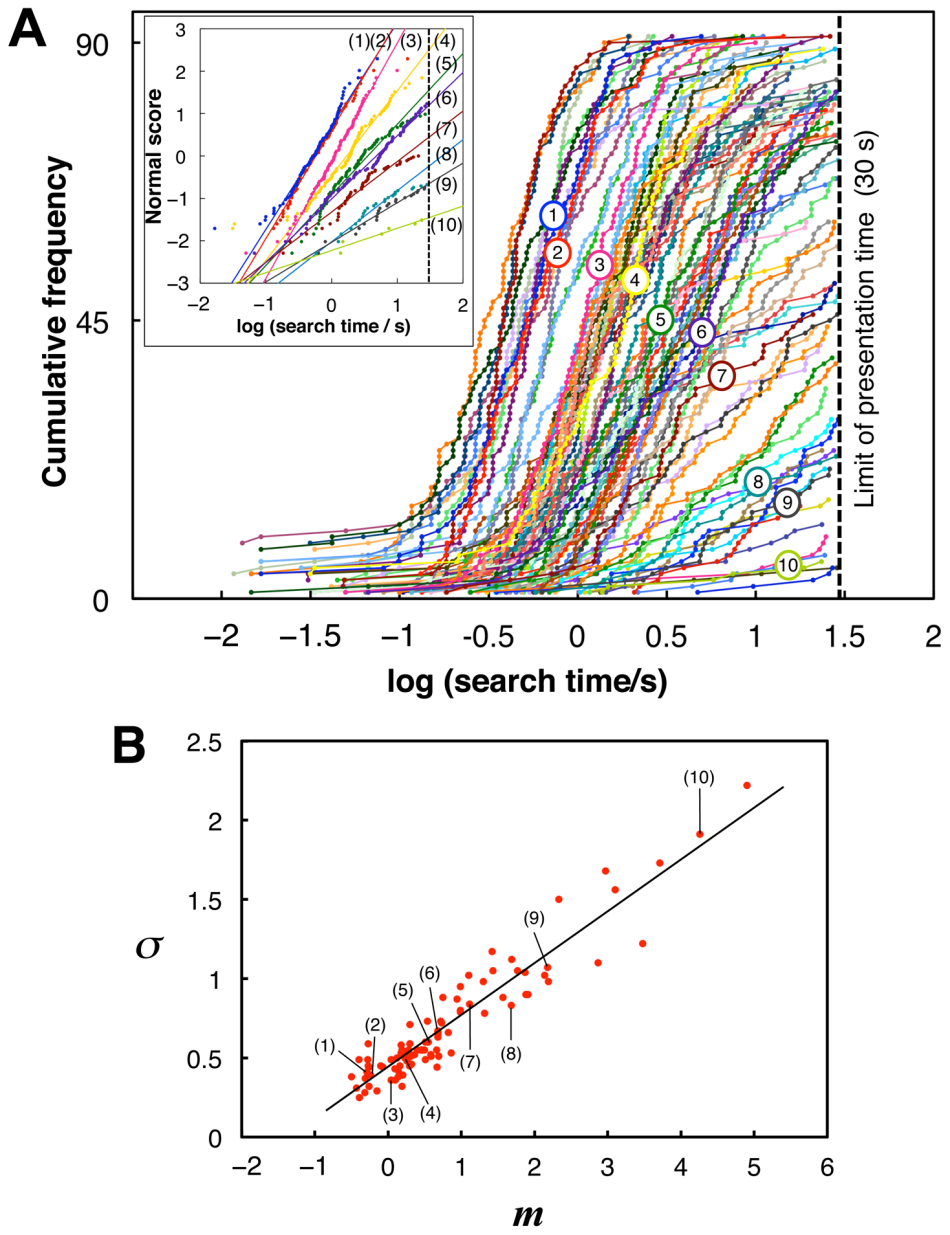
Distributions of search times across subjects. (A) Cumulative distributions of logarithmic search times (obtained within the time limit) across subjects, each of which represents one of the 90 degraded images. Broken lines indicate the presentation time limit (30 s). Numerical labels from 1 to 10 indicate the example stimulus images from Fig. 1. Inset: normal probability plots of logarithmic search times versus normal scores for the example stimuli, each of which is optimally fitted by a line that represents a normal distribution. (B) Relationships between means, *m*, and SDs, *σ*, of the normal distributions fitted to logarithmic search time distributions for all stimuli of degraded images. The linear regression 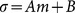 provided the values 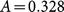 and 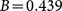. Thus, the time constant 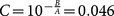 [s] in equation (1).

Fig. 2A shows the cumulative distributions of the search times for all subjects on a logarithmic scale. Distributions for each degraded image were well fitted to a normal distribution (average goodness of fit: *r* ^2^ = 0.97 across images). A highly correlated linear relationship was found between the means, *m*, and standard deviations (SDs), *σ*, of the fitted normal distributions (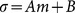, 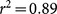; Fig. 2B). The means, *m*, can be reasonably regarded as indicating the difficulty of recognition of each image.

**Figure 3:**
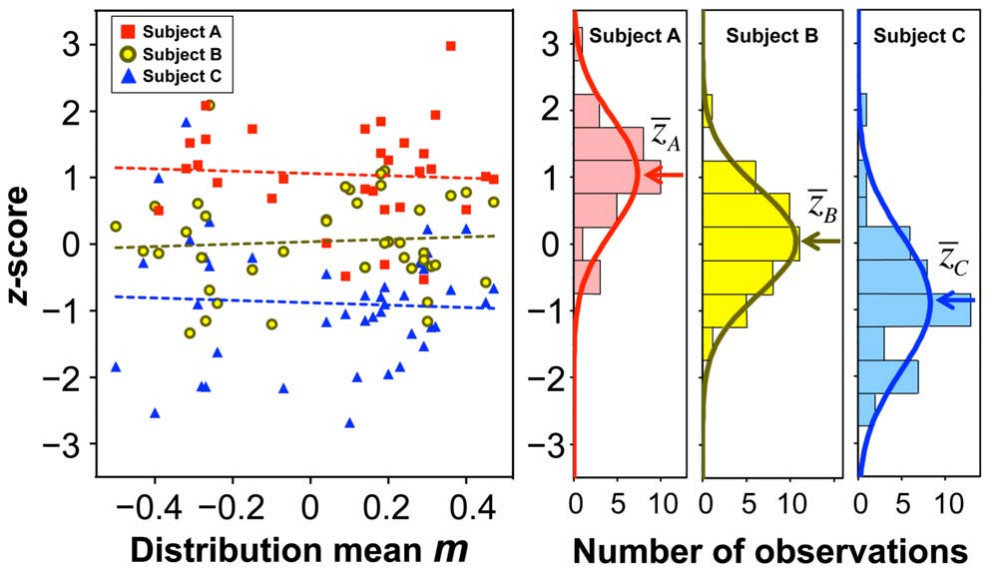
*z*-score distributions of examples of individual subjects. Left panel shows scatterplots of *z*-scores versus distribution mean, *m*, for three typical subjects [the mean *z*-scores of subjects A, B, and C were the fourth highest (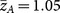), median (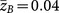), and fourth lowest (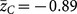) of all subjects, respectively]. Broken lines indicate regression lines of *z*-scores on the distribution mean for each subject. The correlation coefficients for Subjects A, B, and C were −0.06, 0.07, and −0.05, respectively. Right panels show histograms of *z*-scores from these three subjects. Each bar has a bin width of 0.5. Thus, the number of observed *z*-scores (indicated by a bar at *z*_0_) includes 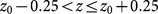. Each curve is a fitted normal distribution (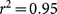, averaged over all 91 subjects). Mean *z*-scores significantly differed between subjects (unpaired, two-tailed *t* tests, 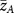 versus 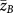: 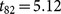, 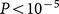; 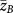 versus 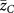: 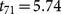, 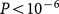).

Individual subject's performance in each trial was rated using a standard score (*z*-score) that represents a standardized value for each subject's logarithmic search time, log *t*, in the distribution for each image: 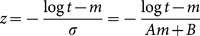. Here the sign is reversed so that a shorter search time corresponds to a higher *z*-score. Fig. 3 shows scatterplots of the *z*-score and the distribution mean, *m*, of three typical subjects. The two parameters are apparently independent. The average correlation coefficient across subjects was 0.09±0.27. (In this paper X±Y always indicates mean ± standard deviation.) The histograms show normal distributions for each subject's *z*-scores. The mean *z*-scores, 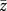, differed significantly among the subjects. Therefore, 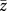 can be considered as indicating each subject's capability to recognize degraded images irrespective of image difficulty. This knowledge of subject-intrinsic capability allows us to express an expected value (i.e., a within-subject intertrial mean) of log *t*, 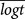, for an image with the mean *m* by using the relation of 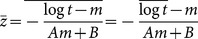, although in actual experiments each image was presented only once to each subject. With simple transformations of 
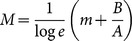
 and 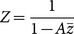, this relationship is converted to a more implicative form:
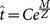
(1)where 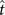 is the mean search time (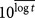 averaged on a logarithmic scale), and *C* is a time constant (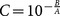). Considering the indications of *m* and 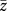 described above, hereafter we refer to *M* and *Z* as “image difficulty” and the “subject's capability,” respectively. Thus, equation (1) indicates that the search time for recognition of a degraded image follows the function of the image difficulty and the subject's capability.

**Figure 4:**
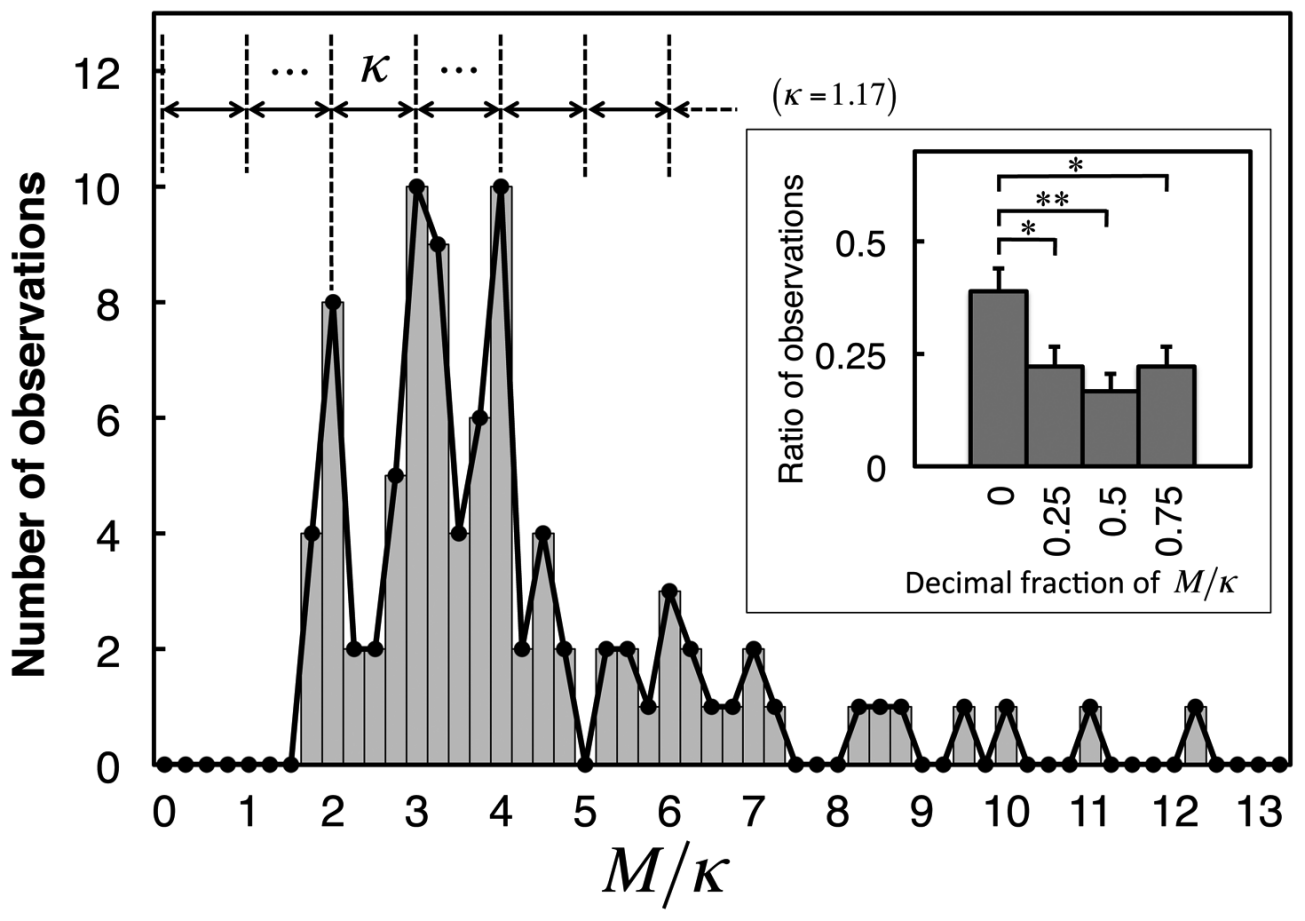
Histogram of image difficulty, *M*, scaled by distribution period, *κ*. The period, *κ*, was determined to equal 1.17 by scanning from 0.5 to 5.0 at 0.01 intervals to maximize the observed frequency of the decimal fraction of *M*/*κ* in the interval 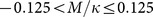. Inset: ratios of observed decimal fractions (error bars indicate standard errors of means (SEM)). The ratio in the quarter interval centered at 0 was significantly larger than those at 0.25, 0.5, and 0.75 (paired, one-tailed *z* test for two population proportions [57], *n* = 90, *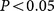, **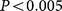).

Furthermore, a periodic distribution was found for image difficulty, *M*, indicating that the difficulty could be scaled by the set of natural numbers (Fig. 4). Thus, the mean search time is given by an exponential function of the natural number that reflects *M* in equation (1). One of the most likely cases in which the time until a certain outcome is given by an exponential function of a natural number is that the outcome is defined by the simultaneous occurrence of multiple stochastic events. As a similar example, the mean number of trials until a gambling machine shows an assigned picture on all reels is given by an exponential function of the number of the reels. Therefore, the present result suggests that the time-consuming process of ER may be theoretically accounted for by a stochastic process model of coincidence of multiple stochastic events, whose number reflects *M*.

### A neurophysiology-inspired theoretical model of emergent recognition

To construct a theoretical model that accounts for the empirical formulation (1) of ER, we considered neural representations of visual objects in the high-level visual system with reference to the knowledge accumulated in neurophysiological studies. The inferior temporal (IT) cortex of human and monkey contains high-level visual areas (e.g., [22]), and neuronal representations of objects in the IT cortex have been well investigated in monkeys. Neurons in the IT cortex selectively respond to particular objects (e.g., a face or a hand) or an object's local features (e.g., a rectangle or a star shape) [23]–[25]. IT neurons with similar selectivity are clustered into columnar structures [25]; an object's image activates multiple neural clusters, each of which represents a visual feature of the object or a spatial relationship between the features [26]–[28]. In particular, neural clusters that responded to certain features were effectively silenced when these features were removed from the images [26]. These findings support the idea of “combination coding,” which proposes that an object is represented by a combination of simultaneously activated neural clusters, each of which represents a visual feature of that object [24], [26]–[29]. Because the removal of local features silences the corresponding neural clusters, it seems to be natural to assume that bottom-up processing of a degraded image of an object fails to activate clusters in the high-level visual system that respond to the features eliminated by image degradation. It is known that even in the absence of selective sensory signals cortical neurons generate spontaneous firings [30]. These firings can be approximated by a Poisson process (i.e., a typical stochastic process of independent discrete events) [31], [32]. Thus it seems to be plausible to presume that neural clusters that are selective to the eliminated features would exhibit stochastic activity despite the absence of the selective sensory signals.

Being inspired by the idea of combination coding of visual objects as mentioned above, we constructed a descriptive theoretical model of ER based on the following assumptions. Recognition of an object hidden in a degraded image is assumed to be due to the combination of components representing the object. The components are assumed to be missing when their corresponding sensory signals are eliminated by the image degradation (hereafter, referred to as “missing components”). We assumed that each of the missing components shows stochastic activation in a Poisson-process manner and that coincidental (i.e., simultaneous in a stochastic manner) activation of all the missing components leads to the complete combination representing the object. Here, for an object in the image, *ν* and *τ* respectively denote the number of missing components of the object and the time interval within which all the *ν* missing components must become active enabling the simultaneous activation required for representing the object. For a given subject, *p* denotes the probability of the Poisson process of activation of a missing component during the interval *τ*. Then the occurrence rate of the Poisson process of effective coincidental activation is given by 
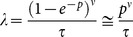
. The search time is defined as the time interval from the onset of steady activation caused by bottom-up processing until the occurrence of coincidental activation. The probability density function of the search time *t* is given by 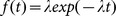 [33]. The mean of log *t* is given by



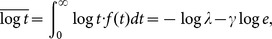
where *γ* is Euler's constant (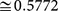) [34], so that an expected value of the search time averaged on a logarithmic scale is given by



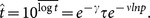



This expression shows the same mathematical structure as equation (1). Thus, we can relate the parameters of the experiment and the model by regarding both the equations as equivalent and noting that *ν* and *p* are given for an image and a subject, respectively:
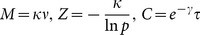
(2)where *κ* is a constant. Because *M* divided by its distribution period ( = 1.17) results in a natural number (Fig. 4), setting *κ* to this period constrains *ν* to the set of natural numbers. In the second expression, letting *p*_0_ denote the probability, *p*, of a subject whose score 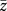 is the population mean (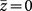, equivalently 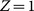), we obtain 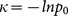, which indicates that for the subject with the mean score, the activation probability of a missing component is given by 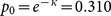 during the interval, 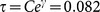 s.

**Figure 5:**
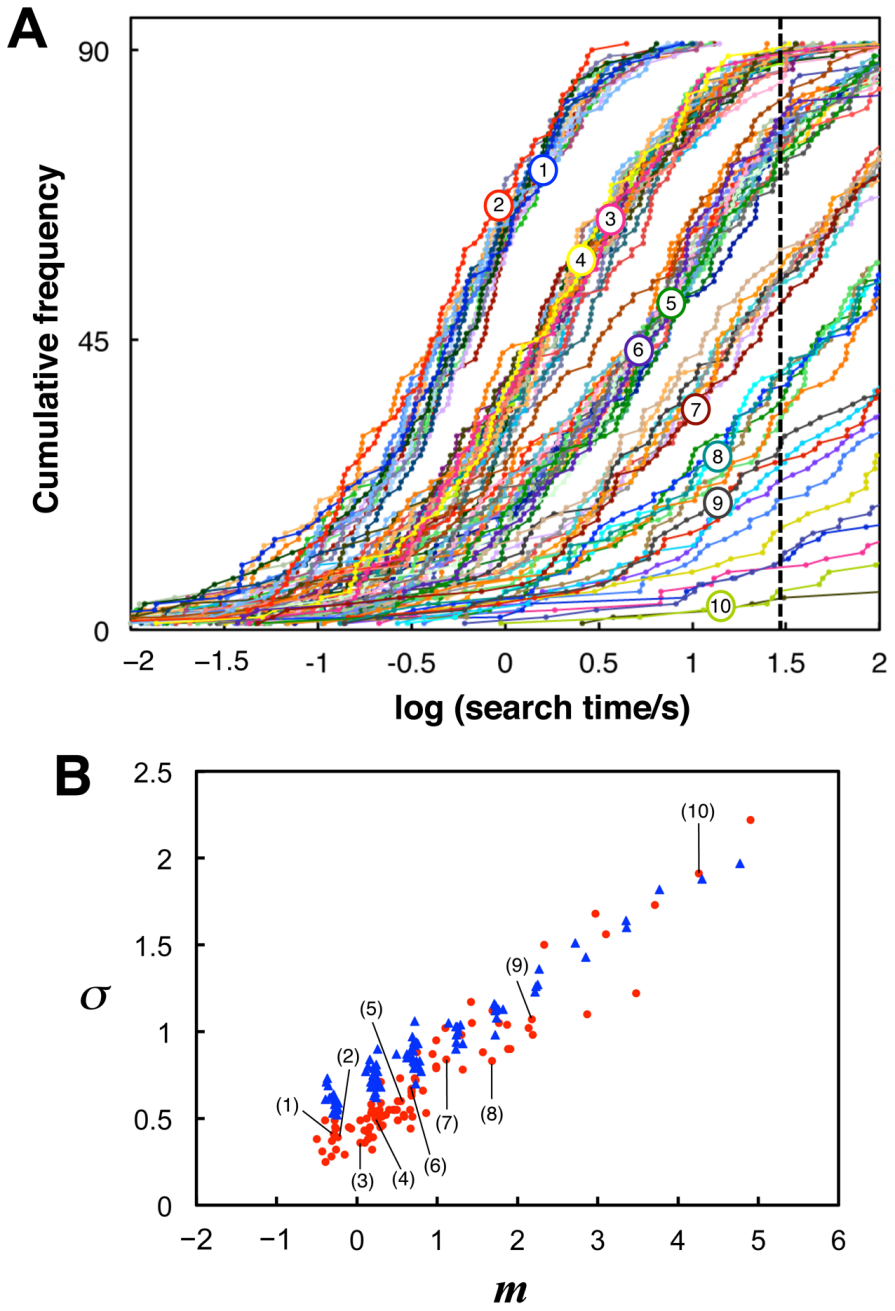
Monte Carlo simulations of the theoretical model. (A) Cumulative distributions of logarithmic search times, defined as the time from search onset until coincidental activation of the missing components (see main text). The numbers of missing components, *ν*, for example stimuli (1) to (10) were 2, 2, 3, 3, 4, 4, 5, 6, 7, and 11, respectively. Broken line indicates the presentation time limit (30 s) used in the experiment. (B) Relationships between means and SDs of logarithmic search time distributions of the model results (blue triangles) in comparison with those of the experimental results (red dots; the same as in Fig. 2B, including labels for example stimuli).

Using values of *ν* (rounded to the nearest natural number) and *p*, which were respectively obtained from the 90 values of *M* of the experiment and from the 91 values of 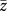 generated with a standard normal distribution, Monte Carlo simulations of the Poisson processes of coincidental activation were conducted. Fig. 5 shows that the model successfully accounted for the characteristics of the experimental results regarding the normal distributions of logarithmic search times (Fig. 5A), the linear relationship between the distribution means and SDs, and the discreteness of the means (Fig. 5B). On the other hand, the model showed slight but systematic differences from the experimental results of the SDs (Fig. 5B). In the next section, we show that these differences in SDs can be eliminated using the model-based corrected method of data analysis, in which the model provides a rationale for evaluating the distribution SD.

### Model-based analysis of search time distribution

In this section, relying on the validity of the model, which was demonstrated by its success in accounting for the characteristics of the phenomenon, the distribution of search times is theoretically analyzed based on the model to find causes of the observed differences in the SDs between the experiment and the model (Fig. 5B). As described above, the probability density function of the search time *t* for a given subject is denoted by 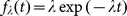, where the subscript *λ* indicates that the rate of coincidental activation is fixed for the subject, and the within-subject (i.e., intertrial) mean of log *t* is given by 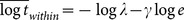. The within-subject variance of log *t* is given by
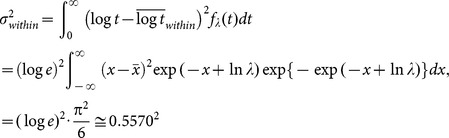
where 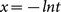 and the integral of the function by *x* gives the variance of a Gumbel distribution, which is a constant regardless of the value of *λ* [35]. As for a population of subjects, where the rate *λ* varies among the subjects, *g* (*λ*) denotes the between-subject probability density function of *λ* in the population. The mean and variance of log *t* observed in the population are given by



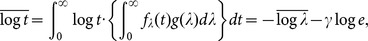
where 
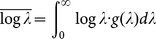
, which denotes the between-subject mean of log *λ*, and



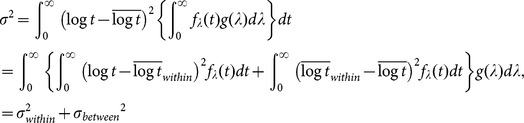
where 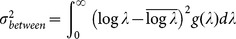, which denotes the between-subject variance of log *λ* in the population. Therefore, the observed variance of log *t* is the sum of within- and between-subject variance as follows:



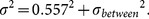
(3)


**Figure 6:**
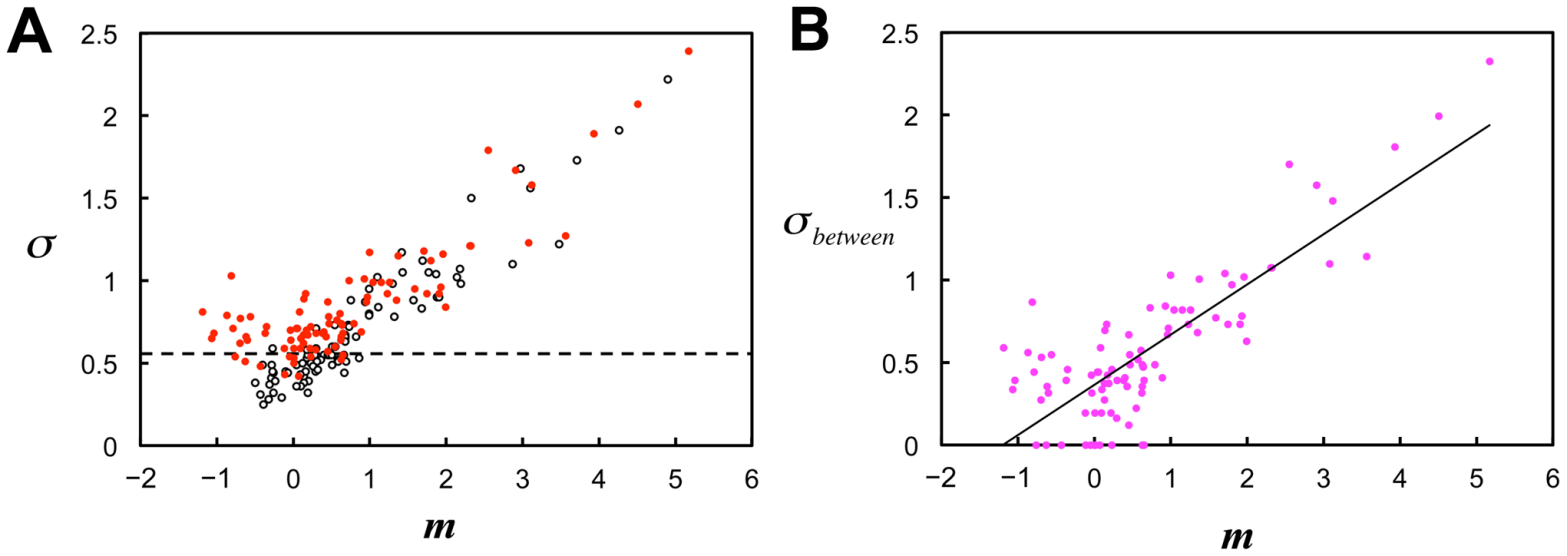
Model-based corrections. (A) Relationship between means and SDs of the distributions of logarithmic search times for all stimuli without and with the model-based interval correction. Original data without the interval correction (open black dots; the same as in Fig. 2B) reveals a substantial number of stimuli with SDs of less than the constant of within-subject SD ( = 0.557) indicated by the broken line, which is the theoretical minimum of the SD. The interval correction using reduction by 300 ms resulted in SDs effectively exceeding 0.557 (red dots) (see Material and Methods for details of the interval correction). (B) Relationship between means and between-subject SDs of the distributions of logarithmic search times. To obtain between-subject SD, *σ_ between_*, the model-based SD correction, 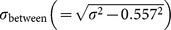, was applied to the observed SD, *σ*, after the interval correction had been applied (see main text for the SD correction). Values of *σ* below 0.557 were converted to 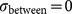 (12% of all data points). The linear regression equation 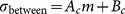, which was applied to all data including those with 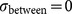, had an *r* ^2^ = 0.68, 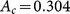, and 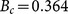.

Although equation (3) requires that the SD observed in the experiment, *σ*, be larger than 0.557, Fig. 6A shows SD of <0.557 for some experimental data, particularly in cases where the difficulty level was low, indicating that the experimental distributions were narrower than the theoretical ones to a certain degree. This discrepancy may have been caused by differences in the estimation of experimental search times for the following reason: we estimated the search time for each degraded image by subtracting the RT for its corresponding color image from that for the degraded image, with the intention of eliminating the time taken for processes common to the two images, thereby, obtaining the search process. These common processes include making decisions on what is being recognized prior to the motor response of key pressing. Recent studies on perceptual decision making have shown that the decision-making process is a separate stage from the preceding sensory processing and subsequent motor response stages. In this decision stage, sensory information is integrated to allow formation of a judgment irrespective of sensory and motor modalities [20], [36], [37]. The rate of decision making depends on the amount of sensory information provided by the preceding sensory processing, taking longer time to integrate poorer sensory information to obtain the amount necessary for making a decision [38]–[40]. In addition, it is shown that the RT required to recognize an unambiguously drawn object in a color image is approximately 100 ms shorter than that required to recognize the same object in a gray-textured image, indicating that sensory information provided in color accelerates decision making of object recognition [21]. Therefore, in the present study, the decision-making stage of original color images might take shorter time than that of degraded images because the sensory processing of color images provided sufficient sensory information to aid in decision making, while sensory processing of degraded images provided only minimal information to represent the object with the help of stochastic activation of the missing components. If this is the case, the search time for a degraded image is estimated to be longer than its true value because the subtraction of RT for its corresponding original image is inadequate to eliminate the entire time taken for processes other than the search process for the degraded image. This leads to a rightward shift of the distribution of search times on a logarithmic scale, and a compression in width, resulting in a smaller SD for the degraded image compared with the true value. Although a detailed investigation of the time required for decision making of the ER task is beyond the scope of this study, we found that reducing the experimental search times by 300 ms effectively improved the SDs such that the results satisfied the model's requirements (Fig. 6A). We refer to this reduction of search time by a certain interval (300 ms) as the model-based “interval correction,” and hereafter we employ the search times to which the interval correction has been applied.

The present model also provides a rationale for the linear relationship between the means and SDs of search time distributions, which is critical for obtaining a consistent relationship between the experiment and the model. Using the above relations, including 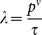, we obtain



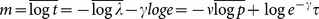
and
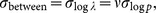
thus
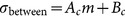
(4)where 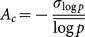, 
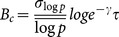
, and the subscript *c* denotes “corrected.” Equation (4) indicates that the between-subject SD, *σ _between_*, has an exactly linear relationship to the mean, *m*. Although the observed SD, *σ*, was empirically found to have a linear relationship to *m* in Fig. 2B, equations (3) and (4) show that this linearity is an approximation of the relation 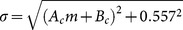, whose slope approaches *A_c_* with increasing *m*. To determine *A_c_* and *B_c_* using the experimental results, we applied a conversion of the experimental values of *σ* (red dots in Fig. 6A) to 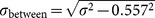 (dots in Fig. 6B), which we refer to as the model-based “SD correction.” The linear regression of *σ_ between_* on *m* provided the corrected values of the parameters of *A_c_* and *B_c_* (Fig. 6B).

**Figure 7:**
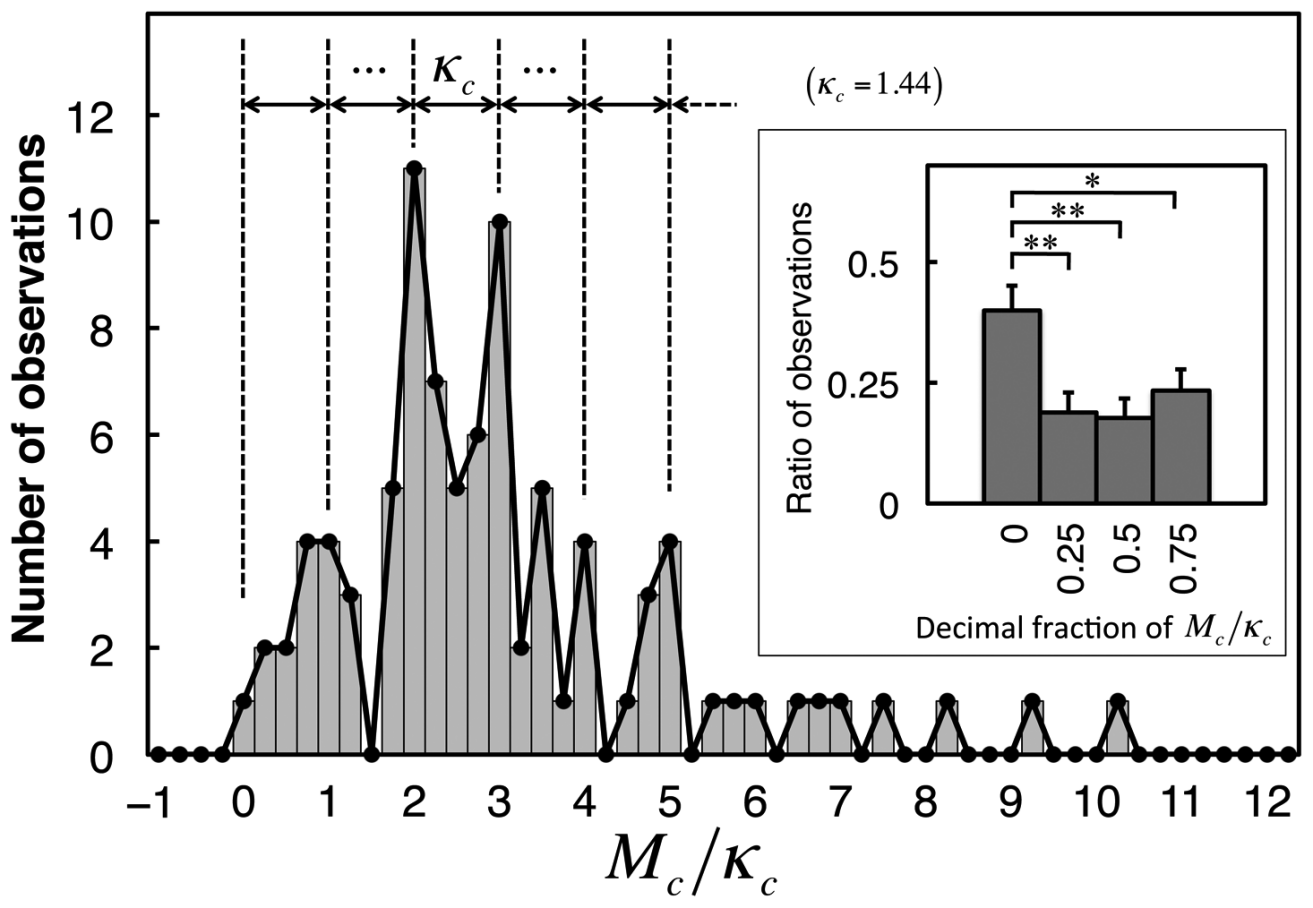
Histogram of image difficulty scaled by distribution period after application of the model-based corrections. The period, *κ_c_*, of the distribution of image difficulty, *M_c_*, was determined to be 1.44 according to the same procedure as that in Fig. 4. Inset: ratios of observed decimal fractions of *M_c_*/*κ_c_* (error bars indicate SEM). The ratio in the quarter interval centered at 0 was significantly larger than those at 0.25, 0.5, and 0.75 (paired, one-tailed *z* test for two population proportions [57], *n* = 90, *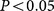, **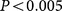). Values of *M_c_*/*κ_c_* were rounded to the nearest natural numbers to be used as the numbers for the missing components, *ν_c_*, in the model. Although the three smallest values of *M_c_*/*κ_c_* were rounded to 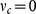, these were regarded as 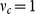 because the search times in case of 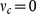 should be zero after application of the interval correction but this was evidently not the case for their corresponding stimuli (Fig. 2A).

**Figure 8:**
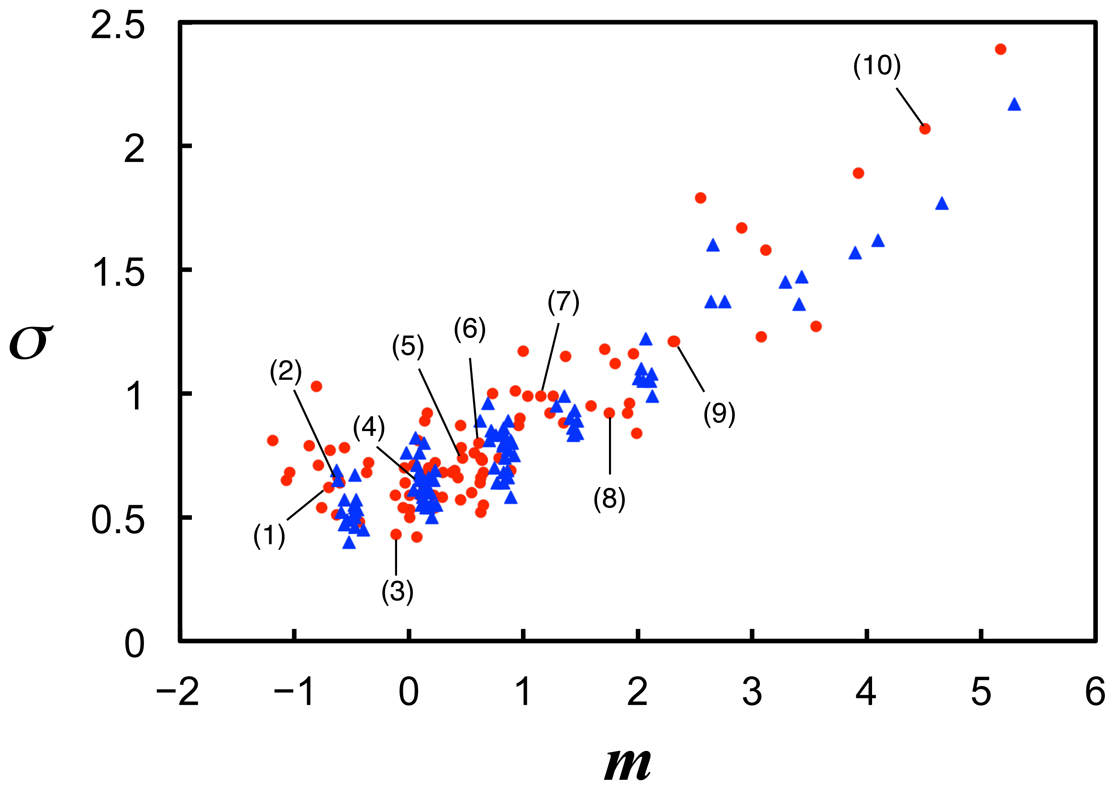
Relationships between means and SDs of search time distributions after application of the model-based corrections. Relationships between means and SDs of logarithmic search times of the experimental results, which are shown by red dots in the same manner as in Fig. 6A with the labels for the example stimuli, and model results, which are shown by blue triangles, after application of the model-based corrections. The numbers of the missing components, *ν_c_*, for example stimuli (1) to (10) were 1, 1, 2, 2, 3, 3, 4, 5, 6, and 9, respectively. The model-based corrections improved agreement between the experimental and model results with the SD differences observed before the corrections (Fig. 5B) being successfully eliminated.

With the SD correction, we obtained a corrected value of a subject's mean *z*-score using *σ _between_* instead of *σ* as follows:



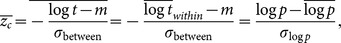
which indicates that the corrected mean *z*-score of a subject is a standard score of the subject's log-activation probability, log *p*. Actually, 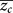, calculated using the experimental values, followed a standard normal distribution (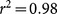; −0.16±0.93, averaged across subjects). Defining the corrected values of *M* as 
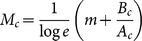
 gave discrete periodic peaks of 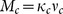, where 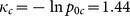 (Fig. 7), offering 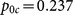. The number of missing components, *ν_c_*, was obtained by rounding the experimental values of *M_c_*/*κ_c_* to the nearest natural number. Using the corrected values of *κ_c_*, 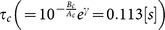, and the sets of 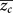 and *ν_c_* obtained above, we conducted Monte Carlo simulations of the Poisson processes of coincidental activation. Fig. 8 shows that the differences in SDs observed between the model results and the experimental results (Fig. 5B) are successfully eliminated using the model-based corrections.

To summarize these results, the model-based analysis improved agreement between the experimental results and the model results, and enhanced the consistency of the explanation in terms of the standard normality of subject's *z*-score distribution and the smallest image difficulty corresponding to one (it was two without the corrections in Fig. 4).

### Comparison with other cognitive phenomenon involving stochastic process

While the present results show that the properties of ER can be explained by the underlying stochastic process model, it is well known that stochastic processes also play an essential role in some other cognitive tasks (e.g., [18]). One of the most intensively studied among these tasks is perceptual decision making (PDM), in which subjects are required to discriminate perceptual stimuli (e.g., degraded object images) as belonging to one of two response categories [20], [36]–[42]. The variation of responses in PDM is not only due to the quality of the stimuli, but also from the intrinsic intertrial variability, leading to a statistical distribution of the RTs and the occurrence of error responses. Such statistical properties of PDM are successfully explained by the Ratcliff drift diffusion model (DDM) [41], [42]. In DDM the decision time is determined as the time for the system state to reach one of the two decision criteria (boundaries) for the responses. The system state is subject to two components of drift and diffusion. The drift component represents the accumulation rate of sensory information of the stimulus, while the diffusion component represents random fluctuations, which give rise to stochastic variations in the accumulation path and time to reach the boundary, and also probabilistic arrivals at the wrong boundary leading to error (i.e., incorrect) responses. The combination of accumulation (i.e., the drift component) and stochasticity (i.e., the diffusion component) makes the DDM explain a wide range of properties observed in PDM (see Materials and Methods for details of the DDM). Since we expect similar general structures of PDM and ER in terms of statistical distributions of RTs, longer RTs for more difficult images, and between-subject variability, we conducted a comparison experiment of PDM and ER in order to see whether alternative explanations similar to the DDM can also be applied to ER (or vice versa) and what characteristics are specific to ER.

**Figure 9:**
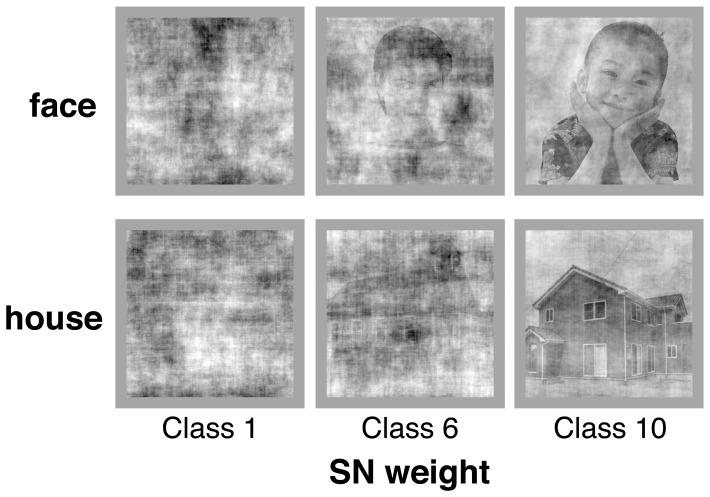
Examples of degraded images used in the perceptual decision making (PDM) section of the comparison experiment. The examples are face and house images of Class 1, 6, and 10 of the signal-to-noise (SN) weight (see Materials and Methods).

Employing another twenty-four subjects, who had not participated in the original ER experiment, we conducted the comparison experiment that consisted of two sections of PDM and ER, in which all subjects participated in this order. The ER section and its data analysis were conducted in the same way as the original ER experiment. In the PDM section we used 200 face images and 200 house images, which were degraded to various degrees by randomizing the phase of their Fourier components according to the weighted mean phase (WMP) technique [43] (see Materials and Methods) (e.g., Fig. 9). Subjects were required to discriminate between face and house images by pressing the assigned keys as quickly as possible while maintaining a high level of accuracy.

**Figure 10:**
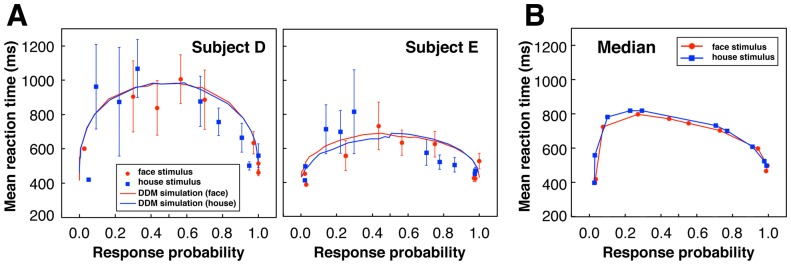
Latency–probability functions. (A) Latency–probability functions (see main text for explanation) of two typical subjects. Mean RT and response probability were calculated in every two classes of the SN weight of stimuli (Class 1 & 2, 3 & 4, 5 & 6, 7 & 8, and 9 & 10). The results for face and house stimuli are represented by the circles and squares respectively, and error bars indicate 2 standard deviations. The continuous lines are results of computer simulations using the drift diffusion model (DDM) (see main text). Red and blue colors respectively stand for face and house stimuli. (B) Latency–probability functions using the median of within-subject mean RT and the average of response probability across subjects.

**Figure 11:**
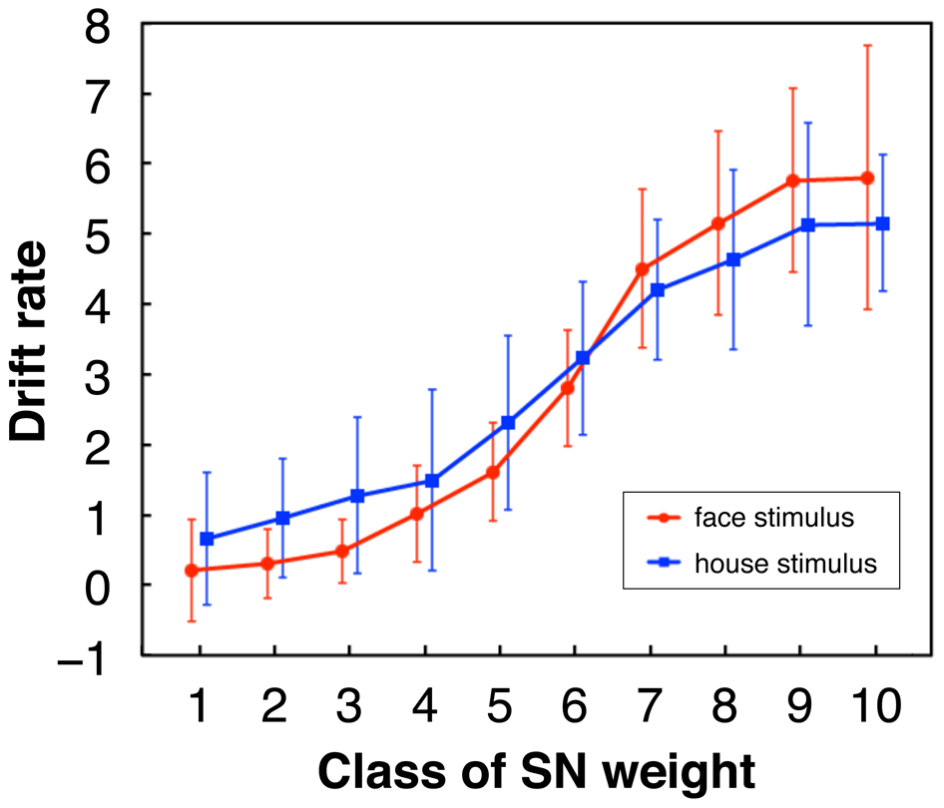
Means of estimated drift rates of the DDM averaged across subjects. Drift rate of each subject was estimated for each class of the SN weight (see Materials and Methods). Error bars represent standard deviation. (All the distributions across subjects passed the Kolmogorov-Smirnov test for normality (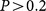).) Although the mean drift rates for house stimuli were estimated to be negative since the decision boundary for house was lower (i.e., at zero) in the DDM, they are plotted with the reversed signs here.

To see whether the experiment of the PDM section captured characteristics of the two-choice PDM tasks established in the previous studies, we obtained latency–probability functions of the data (Fig. 10), in which mean RT is plotted against the response probability (i.e., ratio of correct and error responses) for classes of the signal-to-noise (SN) weight of stimuli (see Materials and Methods for SN weight). This function can be seen as a parametric plot where the varying parameter is stimulus difficulty, that is, class of the SN weight of images here [42], [44], [45]. In this plot, at first the response data are separated into correct and error, and each correct response with probability *p*, which is generally larger than 0.5, has a corresponding error response with probability 1−*p*. As shown in Fig. 10B, many subjects in this experiment showed asymmetric latency–probability functions in which error responses were faster than their corresponding correct responses to easy stimuli and were slower than those to intermediate or difficult stimuli. This result is consistent with the view that the asymmetry of the latency–probability function is a typical property of two-choice PDM, which can be explained by the DDM whose intertrial-variability parameters can be adapted to the asymmetric patterns [42], [45]. We fitted the DDM to the RT data of each subject using the software fast-dm [46] to estimate the parameters of the DDM that explain the empirical data optimally. Fig. 11 shows that the estimated values of drift rates of the DDM increased according to the class of the SN weight of stimuli, demonstrating that reasonable relationships were obtained between stimulus quality (i.e., the SN weight) and drift rate that is a parameter representing mean rate of information accumulation determined by the stimulus quality. Using the obtained values of all the parameters of DDM we performed Monte Carlo computer simulations of the drift diffusion process of DDM to predict RTs and accuracy of each subject. The results showed that the predicted and empirical values were generally close in most subjects (e.g., Fig. 10A). These results indicated that the present experiment captured the primary characteristics known about the PDM tasks.

**Figure 12:**
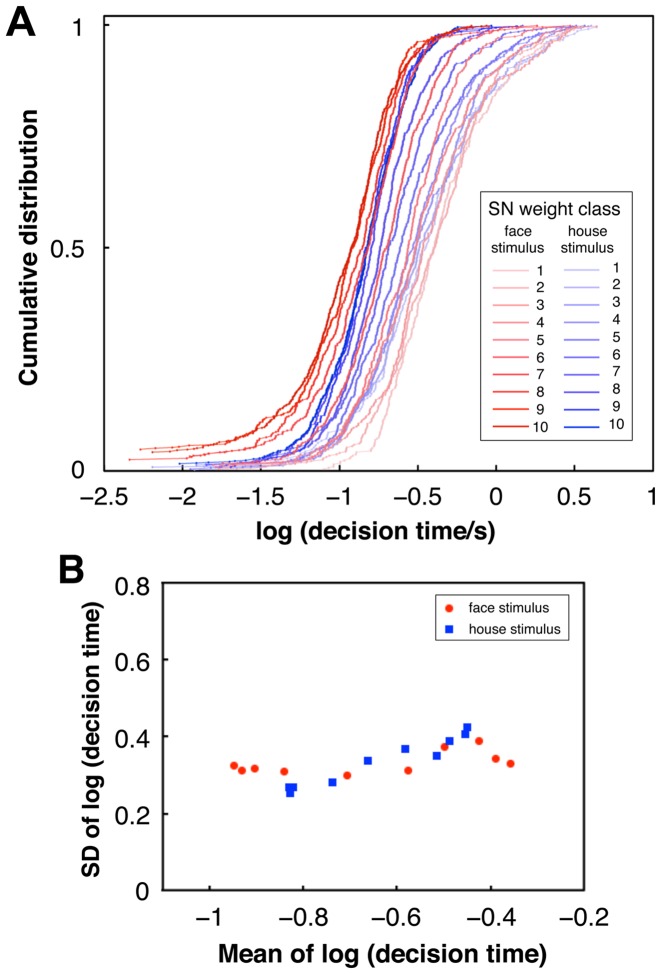
Distributions of decision times across subjects. (A) Cumulative distribution functions of logarithmic decision times over subjects for the 20 classes of the SN weight, each of which includes about 20 images. Each line includes data points whose number is between 206 and 644. (Here, for the graph's clarity we drew 20 lines of the 20 classes instead of 400 lines of all the stimuli, but the statistical tests in the main text were applied to each stimuli.) (B) Relationships between means and SDs of the distributions of logarithmic decision times of Fig. 12A.

To compare PDM and ER in a direct manner, we analyzed the PDM data in the same way as the ER data. In the following analyses we dealt only with correct responses of the PDM task because the error responses are related to the correct responses in a certain manner predicted by the DDM as described above. Since RT of PDM consists of decision and nondecision components, we first subtracted the nondecision component, which was estimated by the DDM fitting (using fast-dm) to data of each subject, from the RT to determine its decision component. For each of the 400 images, the cumulative distribution of the decision times over all subjects on a logarithmic scale was well fitted to a normal distribution (average goodness of fit on a normal probability plot: 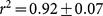 across images) (Fig. 12A). The SN weight of image showed a high correlation with the mean of logarithmic decision times over subjects (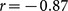, 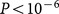 for face images; 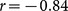, 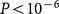 for house images, excluding far out outliers (0.6%)) indicating that the mean logarithmic decision time represents the image difficulty in a similar manner to the ER experiment.

**Figure 13:**
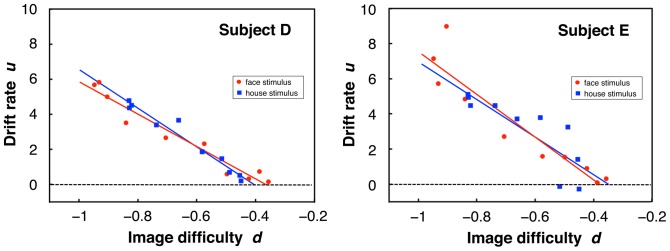
Relationships between drift rate of the DDM and image difficulty for two typical subjects. Drift rates of the DDM were obtained for the SN weight classes of each stimulus category, and image difficulty was defined as the mean logarithmic decision time over subjects (see main text).



The mean and the SD of logarithmic decision times of the PDM, however, did not show a significant correlation for face images (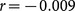, 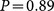) and showed a significant but not strong correlation for house images (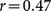, 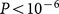) (Fig. 12B). This small dependence of the SD on the mean is a remarkable difference from the result of ER, in which the SD notably increased with the mean (compare Fig. 12 with Fig. 2). In the following, we show that this difference between PDM and ER can be explained based on their theoretical models. Here we refer to the mean of logarithmic decision times over subjects as image difficulty of the PDM task, denoted by *d*. As shown in Fig. 13, the drift rate of DDM depended on the image difficulty in an approximately linear manner for each subject. The average correlation coefficient between drift rate and image difficulty was −0.919±0.076 across subjects and image categories. Because these linear relationships between drift rate and image difficulty showed a narrow range of the intercepts along the image difficulty axis (the average of the *d*-axis intercepts was −0.364±0.077 across subjects and image categories), we regard the *d*-intercept as a constant value of *d*_0_. Then we obtain a linear relation:

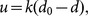
where *u* is a drift rate, 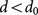, and *k* is a positive constant that depends on subjects and image categories. (The average of *k* was 10.50±2.66 across subjects and image categories.) The mean decision time 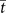 of a subject for an image of difficulty *d* is approximately given by 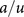, where *a* is the boundary separation of the subject, unless *u* is very small [47], so that we obtain



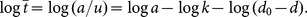



Because *a* and *k* depend on subjects but not on image difficulty, variability of 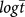 over subjects is constant regardless of image difficulty. This leads to the substantial independence of the SD of logarithmic decision times on the mean, because the difference between the SDs of 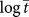 and 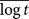 over subjects was negligible (less than 0.03) according to our computer simulations of the DDM for all the subjects. On the other hand, difficulty of the ER stimuli is connected to the number, *ν*, of stochastic events that co-occur to complement missing sensory information (equation (2)). As described above, the co-occurrence of *ν* stochastic events can be modeled by a Poisson process with rate 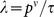, so that the mean of log *t* is given by 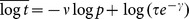, where *p* is the event probability relating to the subject's capability and *τ* and *γ* are constant. Therefore, the between-subject variability of logarithmic time of ER is determined by *ν* times of the between-subject variability of log *p*, leading to the notable dependence of the SD on the mean of the observed distribution. Summarizing the explanation here, the observed difference in the logarithmic time distributions of PDM and ER can be accounted for by the different types of rate-limiting processes underlying the two phenomena: in PDM the stimulus difficulty slows the process in a linear manner while in ER the process is slowed according to the exponential function of the difficulty.

**Figure 14:**
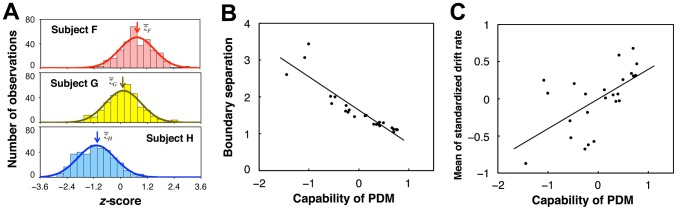
Subject's capability of PDM. (A) Histograms of *z*-scores of three typical subjects whose mean *z*-scores were the second highest (Subject F, 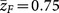), median (Subjects G, 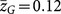), and second lowest (Subject H, 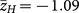) of all the subjects. Each bar has a bin width of 0.3. Each curve is a normal distribution with the same mean and SD as the histogram data. Mean *z*-scores significantly differed between subjects (unpaired, two-tailed *t* tests, 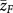 versus 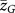: 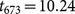, 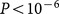; 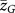 versus 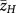: 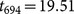, 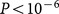). (B) Relationship between subject's capability of PDM and boundary separation. The capability was defined as the mean *z*-score of the subject (see main text). (C) Relationship between subject's capability of PDM and within-subject mean of drift rates each of which was standardized over subjects in a stimulus class of the SN weight.

**Figure 15:**
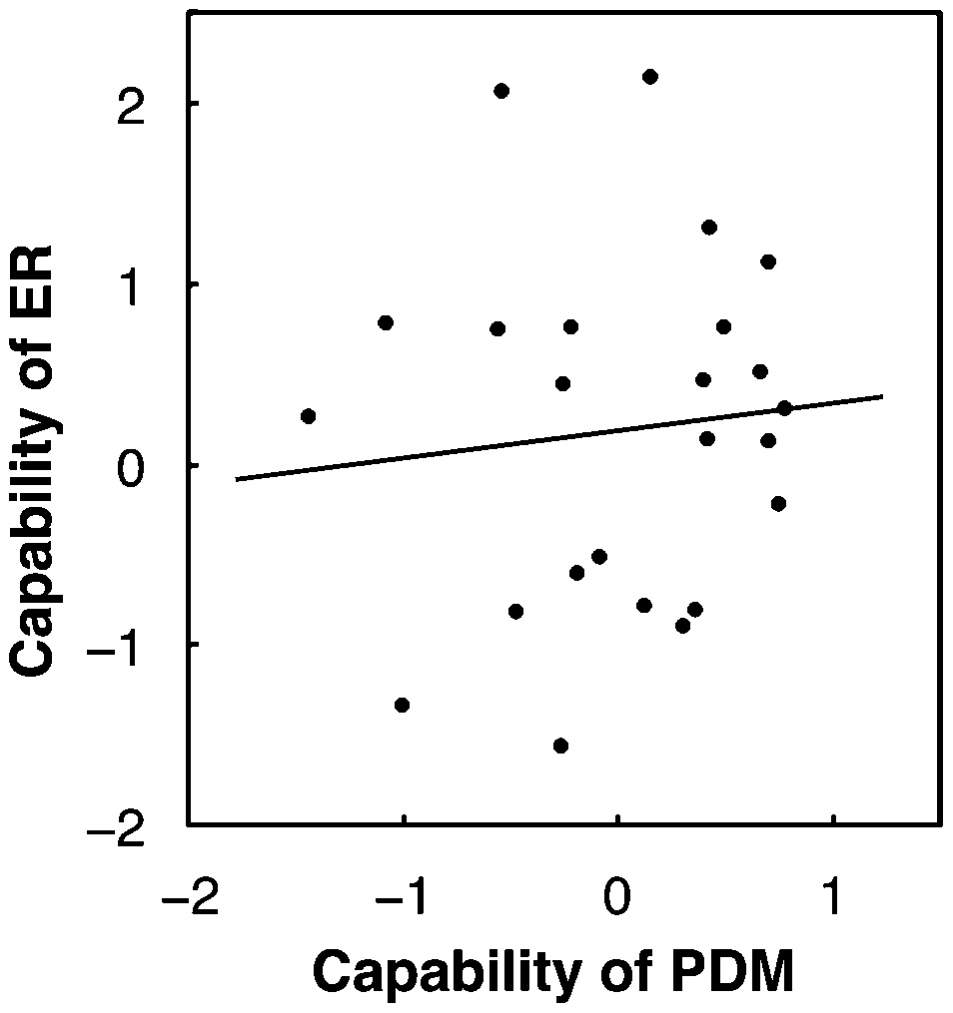
Scatterplot of capability of PDM and capability of ER over subjects. Data were obtained from twenty-four subjects who participated in both PDM and ER experiments (see main text).

To quantify between-subject variability of PDM, we adopted *z*-scores in logarithmic decision time distributions over subjects following the ER analysis. (The sign of *z*-score was reversed so that a shorter decision time corresponds to a higher *z*-score.) We found that a subject's *z*-score was independent of the image difficulty because the correlation between a subject's *z*-score and the mean of the logarithmic decision time distribution was so low as −0.002±0.132 averaged across subjects. The within-subject mean *z*-scores differed significantly among subjects (Fig. 14A). Thus we defined a subject's capability of PDM (with regard to the degree of quickness) as the mean *z*-score in a similar way to subject's capability of ER. The capability of PDM was correlated strongly with two parameters of the DDM: the response-boundary separation (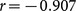, 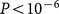) (Fig. 14B) and the within-subject mean of drift rates standardized over subjects (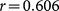, 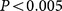) (Fig. 14C), indicating that subjects with smaller boundary separation and larger drift rate have larger capability. Thus, this capability effectively captures between-subject variability in quickness of the PDM in a manner consistent with the DDM framework. Since all subjects of the PDM experiment also participated in the ER experiment, we obtained their capabilities of ER (with the model-based corrections). As shown in Fig. 15, the capability of ER had no correlation with the capability of PDM (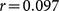, 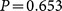) (not with either boundary separation (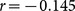, 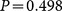) or mean standardized drift rate (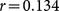, 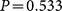) of the DDM). The results suggest that neural modules associated with the rate-limiting processes of PDM and ER may be different although both functions are related to visual recognition of degraded images.

To summarize the comparison experiment, the results showed remarkable differences between PDM and ER in the properties of time distributions and the subjects' capabilities, suggesting that fundamental stochastic processes of the two phenomena are of different types and that neural modules determining rate-limiting stages may be different. In contrast to PDM, ER was characterized by an exponential increase of time according to the image difficulty, which covered a wide range of RTs from less than one second to tens of seconds.

## Discussion

The present study of ER shows that the time-consuming process of recognizing an object in a degraded image without any prior top-down information follows an exponential function related to two independent parameters: image difficulty and subject's capability. This function was successfully accounted for by the stochastic process model in which coincidental activation of the missing components of an object leads to the complete representation of the object. The agreement between the experiment and the model was improved by the model-based corrections of data analysis, supporting the notion that the stochastic process is the underlying mechanism of ER. To see whether the present results were specific to the ER task, we conducted the comparison experiment in which the PDM task, which is known to involve a certain stochastic process, was compared with the ER task. The results showed that stimulus difficulty slowed the process in a linear manner in PDM in contrast to exponential dependence in ER. The exponential time can cover a very wide range of RTs from tenths to tens of seconds, which seems to be specific to ER. Under situations requiring ER, it seems to be a reasonable strategy for the visual system to keep every possibility open in attempting to identify an obscure object by employing nonselective stochastic neural activation that may match the deficient input pattern of the object by coincidence.

A study of functional magnetic resonance imaging in humans showed that the timing of recognition of Mooney face images without prior exposure to undegraded versions correlated with the activity in face-selective areas in the high-level visual cortex, but not with that in the frontal or parietal areas, suggesting that face-selective areas may play an important role as a rate-limiting step of Mooney image recognition in the absence of sufficient top-down information [4]. This result is consistent with the present study with respect to the critical role of the high-level visual representations as a rate-limiting step of the ER. This result seems to be in contrast to recognition facilitated by prior top-down information, in which the frontal and parietal areas also play important roles [6], [7], [13], [16].

The hypothesis that stochastic activation of high-level visual system causes ER may raise the question of why arbitrary neural activation does not normally lead to visual hallucination of objects (i.e., perception accompanied with the reality of objects that do not exist) in the absence of sensory stimuli. With regard to visual perception with reality, studies using transcranial magnetic stimulation have shown that disruption of the activity of the primary visual area V1, even after activation of high-level visual areas, impairs visual awareness of percepts [48] or perceptual clearness of objects [49], [50]. Physiological studies have shown that feedback signals from high-level visual areas to the visual area V2 lacking feedforward signals from V1 cannot drive neurons in V2 [51], an area responsible for representing illusory contours. These contours may be critical for recognizing degraded images. These findings regarding the critical role of V1 suggest that object perception with reality requires V1 activity to be driven at least partly by the sensory stimulus of the object. Then, recurrent interactions between V1 and the higher areas, which are thought to integrate complementary information of spatiality of an image held by V1 and high-level representations into conscious perception [52]–[55], may play an important role in selecting high-level activation that can be compatible with V1 activity driven by sensory signals of a degraded image in a manner that prevents hallucinations.

## Materials and Methods

### Ethics Statement

This study was approved by the Ethics Committee for Human and Animal Research of the National Institute of Information and Communications Technology, Japan. All subjects gave prior written informed consent in accordance with the guidelines approved by this committee.

### Subjects

Ninety-one healthy subjects (59 males and 32 females), aged 21.7±1.8 years, participated in the (original) emergent recognition (ER) experiments. (In this paper X±Y always indicates mean ± standard deviation.) All subjects were recruited from academic environments and were paid for their participation. Each subject had normal or corrected-to-normal vision and passed the tests of Visual Object and Space Perception Battery (Thames Valley Test Company, Bury St Edmunds, Suffolk, England). (Subjects of the comparison experiment are described in the dedicated section below.)

### Stimuli

Ninety digital color images were selected from clip-art collections (MasterClips Image Collection (IMSI, Novato, California, USA), Photo Mantan (DesignEXchange, Tokyo, Japan)) and personal photographs (e.g., see Fig. 9). Each image contained a distinct object that could be easily described verbally (e.g., “a dog lying with its head turned to the left”). In the original image session (described below), all subjects were able to identify the object in each image correctly (with very few exceptions) with short reaction times (RTs) of 0.77±0.42 s (averaged over stimuli and subjects). Thus, all subjects had knowledge of all the tested objects, and all original images contained sufficient information for the objects to be recognized. To avoid any interference due to categorical bias, images of objects from various categories were selected: 5 were images of human figures, 11 of domestic mammals, 17 of wild mammals, 11 of birds, 9 of other animals, 5 of flowers, 11 of tools or foods, 12 of transportation, and 9 of natural or architectural scenes.

These color images were degraded by monochromatic binarization. A binarization threshold was set for each image manually using Photoshop (Adobe Systems, San Jose, California, USA) with the intention of distributing the subjective difficulty of the degraded images within a recognizable range. Because the binarization threshold for each image was arbitrarily determined on a 256-step gray scale, this degradation process was unlikely to cause any artifactual discreteness of the image difficulty found in this study (Fig. 4 and Fig. 7). Subjects were seated 100 cm from a CRT monitor on which the stimulus images were presented subtending 10° of visual angle on the longer sides (573 pixels) with luminances of 40 and 0 cd·m^−2^ in the white and black regions, respectively. The images were presented on a black background.

### Experimental sessions

Experiments were conducted with one subject at a time in a dark soundproof room. They consisted of four separate sessions in the following order: practice, degraded image viewing, original color image viewing, and simple RT measurement. In the practice session, subjects experienced recognition of an object hidden in a sample degraded image and were asked to be sure of their recognition of the object referring to its original color image. Then, the instructions were provided about the tasks for degraded images and color images (as described below), and several practice trials were conducted using degraded and original images specific to the practice session. In the sessions of degraded image viewing and original image viewing, each image was used in a single trial with a random turn. Each experiment required about one hour for completion including short breaks provided between the sessions.

### Tasks

For both degraded and original images, subjects were instructed to gaze at a fixation point centered on the monitor until the image was presented in order to control for their initial observation. Free eye movements were allowed after the presentation of each image. Subjects were required to press a key as quickly as possible when they recognized something meaningful in the stimulus image. The image was turned off immediately after the key was pressed, and the RT was recorded by a computer as the time interval between the stimulus onset and the subject's key press with a precision level of 30 ms. Subjects were then asked to report verbally what they had recognized to the experimenter. Although degraded images may have multiple interpretations (like the vase and faces in Rubin's ambiguous figure [9]), only the object shown in its original image was accepted as correct. Subjects were encouraged to be as descriptive as possible. Similar but distinct answers such as “a small animal like a squirrel” and “a prairie dog” that described the correct object were equally acceptable. When answers were unclear, the experimenter prompted the subjects to add more detail in order to confirm recognition (e.g., “Please describe the position and posture of the object that you saw in the image”). Subjects were informed if their answers were correct. When the correct answer was provided, the next trial was conducted. When an incorrect answer was provided, in order to prevent hesitation, repeated observations of the same image were allowed until the accumulated presentation time reached the time limit. The time limit was set to prevent trials of long duration. Each image was presented for a maximum of 30 s, after which the image was turned off without revealing the correct answer and the next trial followed. For each image, at least five of the 91 subjects achieved correct answers within this time limit. Only 4% of all the successful trials of degraded images were obtained from the repeated observations, and we included these data in the further analyses because eliminating them had no qualitative influence on the results of this study.

In the session of simple RT measurement, a white disk was briefly presented 30 times at random intervals, and the subjects were required to press the key as quickly as possible when they saw the disk. The grand mean value of simple RTs was 0.26±0.04 s (averaged across subjects). No correlation was found between mean simple RTs and mean *z*-scores, 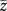, of subjects (two-tailed *t* test for correlation: 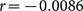 across subjects, 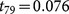, 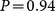), indicating that the subjects' capabilities were not influenced by their scores of simple RT.

### Normal probability plot of logarithmic search times

For each degraded image, a normal distribution was fitted to the distribution of logarithmic search times on a normal probability plot (Fig. 2A, inset) using the least squares method excluding far out outliers that were larger than the upper quartile plus 3 times the interquartile range (6.6% on average). The *z*-score of the logarithmic search time was given by the inverse function of the standard normal distribution whose probability was 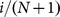, where *i* is the ascending ordered rank of the logarithmic search time and *N* is the total number of subjects (*N* = 91). Negative search times were given by a very low percentage (1.6%) of correct answers mostly for the easiest degraded images. For example, RTs for a degraded image and its original image were as short as 0.65 s and 0.80 s, respectively, indicating that their difference was critically influenced by fluctuations in RT. These cases did not provide logarithmic values, but were counted as the smallest values in the cumulative distributions.

### Subject's capability

Subject's capability, *Z*, was determined by using the definition, 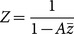, where 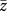 was obtained by averaging *z*-scores for each subject. Because *z*-scores were obtained only for correct answers achieved within the time limit (30 s), difficult degraded images provided only high values, causing a bias in the *z*-scores toward higher values. To avoid this bias, *z*-scores for the 43 easiest degraded images were used for determining the averages. For these 43 images, at least 85% of subjects achieved correct answers within the time limit. The mean of 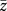 for all the subjects was close to zero (0.03), which shows that the bias was minimal. In addition, 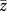 followed a normal distribution (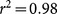). Although it was based on only the 43 easiest images, 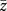 was effectively applicable to all 90 images; for the other 47 images, an adequate ratio (95.1%; averaged across subjects) of logarithmic search times was observed within the 95% prediction intervals of log *t*, which were calculated from the 95% intervals of the *z*-scores.

To obtain a subject's mean *z*-score with the model-based corrections, 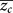, the corrected *z*-scores, *z_c_*, of each subject were selected for the 50 easiest degraded images to avoid the bias. Then, *z_c_* for the five easiest images were excluded from the averaging because they showed very large fluctuations due to extremely small *σ _between_* as the denominator in obtaining the corrected *z*-score by 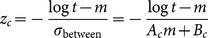 (see Fig. 6B). The corrected *z*-scores for the remaining 45 images were averaged to determine the corrected value of a subject's mean *z*-score, 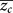.

### Monte Carlo simulation

The time until coincidental activation of *ν* missing components of a subject with mean *z*-score 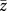 was calculated by computer simulation using Matlab (MathWorks, Natick, Massachusetts, USA). Given the parameters *τ* and *κ* (see main text), a Poisson process with a rate of 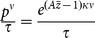 was simulated using the Monte Carlo method, in which the occurrence of an activation over a time step of 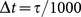 with the probability 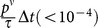 was determined using random numbers. When the first Poisson event occurred at the j-th step, the corresponding search time was given by 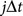.

### The model-based interval correction

Based on the argument that experimental search times may be longer than their true values in the first analysis of the original ER experiment, resulting in their narrower distributions on a logarithmic scale (see main text), the effects of reducing search times to increase their SD were investigated. According to a previous study of image discrimination, times required for decision making could be shorter by approximately 300 ms to 400 ms for easy tasks than for difficult tasks [40]. Therefore, reduction times of 0 (i.e., no reduction), 100, 200, 300, and 400 ms were applied to all search times obtained in the experiment. The results showed that for each of the respective reduction intervals, the numbers of stimuli with SD of <0.557, which is the theoretical minimum of SD, were 46, 34, 19, 11, and 5, indicating that the reduction of search times effectively increased the SDs. In order to determine the appropriate reduction interval, these numbers were compared with the expected values obtained in the Monte Carlo simulations of our model. The simulations showed that SDs of <0.557 were possible for a certain ratio of stimuli owing to statistical fluctuations in the Poisson process that occur when a finite number of subjects (*n* = 91) is tested. The simulation results (averaged over 1000 repetitions) showed that the ratios of stimuli with SD of <0.557 were 38.1%, 5.2%, and 0.0% for the number of missing components of *ν* = 1, 2, and 3 or greater, respectively. Using these ratios and the number of *ν* values observed in the first and second peaks of their distribution, which were respectively 16 and 25 (Fig. 4), the expected number of stimuli with SD of <0.557 was estimated to be 7.4, which was closest to and not more than the experimental number with a reduction interval of 300 ms (11). Using 300 ms as the reduction interval, the interval correction together with the SD correction effectively improved agreement between the experimental and model results (see main text). As a result, the observation frequencies of 1 and 2 of *ν* obtained after application of the model-based corrections (Fig. 7) were respectively equal to those of 2 and 3 of *ν* in the first analysis (Fig. 4), showing that this technique allowed the discrete structure of the distribution of *ν* values to be maintained.

### Comparison experiment

For the comparison experiment we employed twenty-four healthy subjects (14 males and 10 females), aged 22.1±2.1, who had not participated in the original ER experiment. The same procedures as the original ER experiment were applied to all subjects: receipt of the written informed consent, test of basic visual functions, and payment for participation. The comparison experiment consisted of two sections of perceptual decision making (PDM) and ER, in which all subjects participated in this order. The ER section and its data analysis were conducted in the same way as the original ER experiment.

For stimuli of the PDM section, we used a set of 235 face and 235 house grayscale images, of which 35 face and 35 house images were used for practice and 200 each for the test sessions. The original images were obtained from clip-art collections (Sozaijiten (Datacraft, Sapporo, Japan) in addition to the collections described above) and from various sites on the web. They were cropped to 512×512 pixels in size, and converted to 8-bits/pixel gray-level depth. Fast Fourier transforms (FFT) of these images were computed to produce their magnitude and phase matrices. The phase matrix of each image was modified using the weighted mean phase (WMP) technique [43], [39], in which a vector of length *w* (in the range of 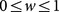) and phase of the image was combined with another vector of length (1−*w*) and phase of a uniform random noise in the range of 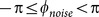 to generate a vector having the *w*-weighted mean phase with the random noise. A different noise phase was used for each value in the phase matrix under a fixed *w* for the image. For the magnitude components, the average magnitude matrix across all the 400 original images (for the test sessions) was stored, and the magnitude matrix of each image was linearly combined with the average matrix using the same weight *w* as the phase matrix of that image. Stimulus images were produced by the inverse FFT of these modified magnitude and phase matrices. Thus the parameter *w* represents the signal-to-noise (SN) weight of the original image, or the inverse of the image degradation level. The SN weight of each image was a uniformly distributed random number between 0.2 and 0.6 to see if any discreteness in the task difficulty like the ER experiment was detected under the continuous distribution of image degradation levels. (As a result of the experiment, we could not detect any discreteness of difficulty of the PDM task.) We defined the SN weights of 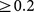 and 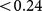 as Class 1, and in the same manner, ranges of the SN weights, [0.24, 0.28), [0.28, 0.32), [0.32, 0.36), [0.36, 0.40), [0.40, 0.44), [0.44, 0.48), [0.48, 0.52), [0.52, 0.56), and [0.56, 0.6] as Class 2 to 10, respectively. Each class of face and house stimuli included 20±4 images. All the images were normalized by the average luminance (11 cd·m^−2^) and RMS contrast (9 cd·m^−2^), and were surrounded by a gray background of the average luminance. Stimuli were presented on the same CRT monitor at the same distance as the original ER experiment (resulting in an image size of 9°×9°) using the software Presentation (Neurobehavioral Systems, Berkeley, California, USA).

The PDM section consisted of sessions for practice and test, in which 70 and 400 different images were respectively used. Each image was presented in a single trial in a randomized order. On each trial the stimulus image was presented after a fixation point displayed on a gray background of average luminance for a random time interval (1000–2000 ms). Subjects were required to discriminate between face and house images by pressing the assigned keys as quickly as possible while maintaining a high level of accuracy. The presentation was terminated by the subject's key press. A feedback message was presented for 500 ms following each response to indicate whether the response was correct or error. The instruction did not emphasize particularly on speed and accuracy of performance (i.e., no speed-accuracy manipulation type) [42]. (When subjects did not respond within 5 s, a “too slow” message was indicated, but these occurred only in 0.02% of all the trials.)

**Figure 16:**
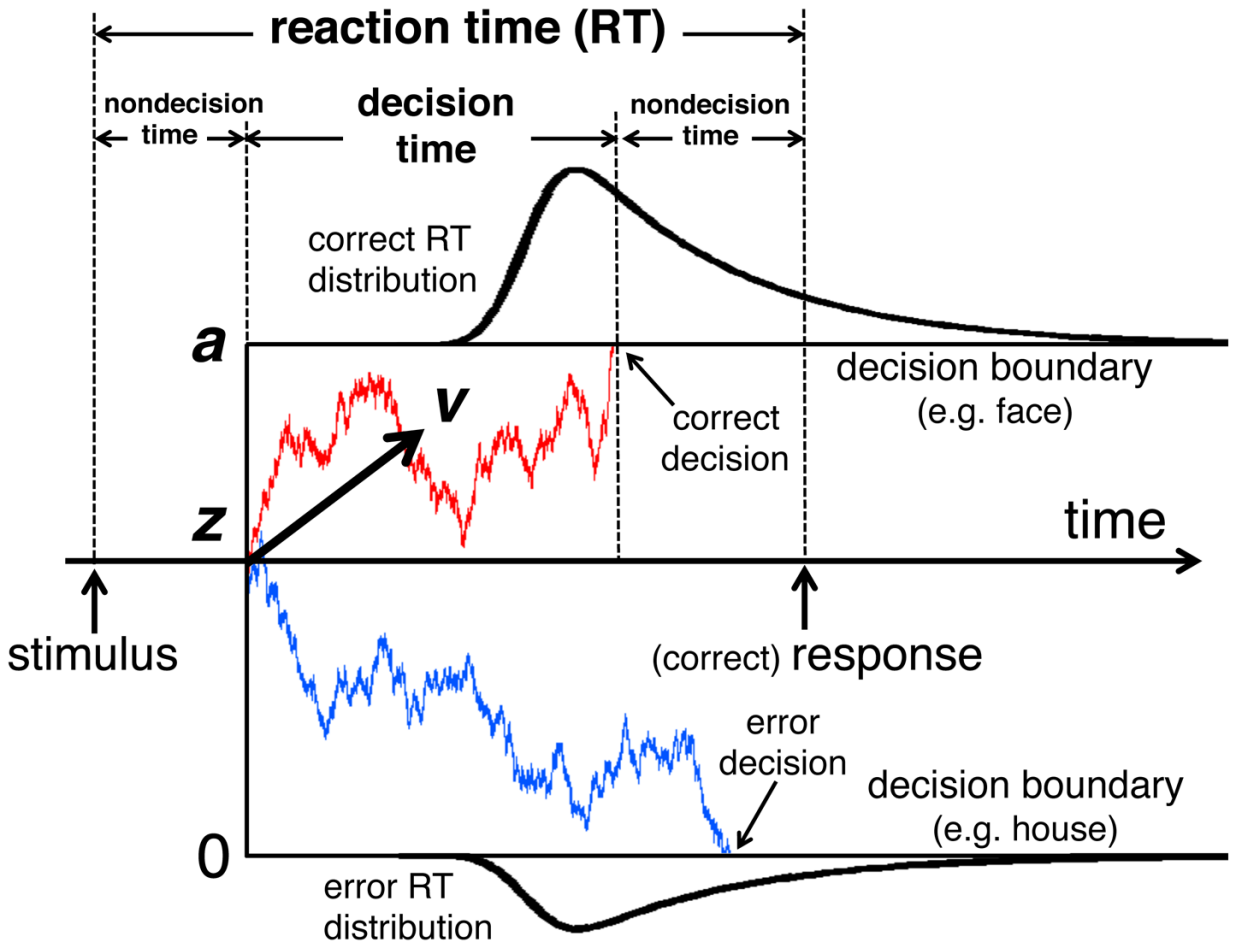
The drift diffusion model (DDM). The vertical axis represents the accumulation of sensory information. The nondecision time, *t*_0_, consists of two components before and after the decision time. For other parameters and details of the DDM, see Materials and Methods. Two simulated paths are drawn with a positive drift rate, *u*, which means that a face response is correct in this example. The RT is indicated for the correct response.

The drift diffusion model (DDM) [41], [42] is a theoretical model which is known to be successful in explaining a wide range of observed relationships between RTs and accuracy of two-choice PDM tasks [20], [42], [45]. As illustrated in Fig. 16, the DDM assumes that noisy information about the stimulus is accumulated over time from a starting point, *z*, toward one of the two decision boundaries for the responses and that a decision is formed once the boundary has been reached. The mean rate of information accumulation is represented by the drift rate, *u*, which is determined by the quality of information that is extracted from the stimulus. The boundary separation, *a*, represents the distance between the two decision boundaries. Larger values of *a* indicate that more information must be accumulated to make a decision. The starting point, *z*, represents the initial position between the two boundaries, representing the subjects' a priori bias for one of the two alternatives by the degree of deviation from *a*/2. The nondecision time, *t*_0_, quantifies the duration of sensory and motor processes that are unrelated to the decision process. In addition to these principal parameters, the model assumes parameters quantifying intertrial variability in the drift rate (normal distribution with mean *u* and standard deviation *s_u_*), the starting point (uniform distribution around *z* with range *s_z_*), and the nondecision time (uniform distribution around *t*_0_ with range *s_t_*_0_). The parameters of *s_u_* and *s_z_* are particularly important because they enable the DDM to predict the asymmetric latency–probability functions (see main text) [42]. The noise magnitude in the model is defined by intratrial variability of the drift rate, which brings about stochasticity in the information accumulation process and is modeled by a Wiener process that is a stochastic process of diffusion (see the next paragraph). The magnitude of this variability, *s*, is a scaling parameter of the DDM and is set a priori to a fixed value, which was 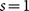 in the present study. All parameters (except for *t*_0_ and *s_t_*_0_) are identified by ratios to this scaling parameter. We fitted the DDM to the experimental data of each subject using the software fast-dm developed by [46] (version 30, available from the website: www.psychologie.uni-heidelberg.de/ae/meth/fast-dm). Fast-dm estimates all parameters of the DDM from the empirical RT distributions of two categories of responses, by means of efficiently computing the cumulative RT distributions predicted by DDM parameters [56] and minimizing the Kolmogorov–Smirnov statistic that is the maximal distance between the predicted and the empirical cumulative RT distribution. In the present estimation we assumed that drift rate depended on stimulus difficulty, thus requiring separate values for drift rates of the ten classes of the SN weight of each stimulus category, whereas all other six parameters were assumed to be constant within a subject. The parameter of calculation precision was set to 3.0 (default value of fast-dm). The estimated values of parameters averaged across subjects were: 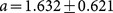, 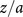 (starting point relative to *a*) 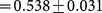, 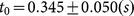, 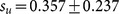, 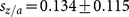, and 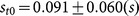. As for *u* (drift rate), see Fig. 11. (All the parameter distributions passed the Kolmogorov-Smirnov test for normality using Matlab (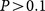).)

Using the estimated values of parameters of DDM, we performed Monte Carlo computer simulations of the drift diffusion processes of the DDM. For each subject we set *a* and *t*_0_ to the estimated values, and the starting point in each trial was selected randomly from a uniform distribution whose center and range were the estimated values of *z* and *s_z_* respectively. We varied the mean drift rate *u* over an appropriate range, and for each value of *u* we repeated 20,000 trials, in each of which an actual value of drift rate, 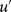, was selected randomly from a normal distribution with mean *u* and standard deviation of the estimated value of *s_u_*. The drift diffusion process was simulated using the Monte Carlo method, in which the displacement on the information accumulation axis over a time step 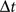 was the sum of the drift component of 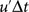 and the diffusion component of a random number from a normal distribution whose mean was zero and standard deviation was 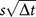 (displacement of a Wiener process) [47]. We set 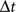 to 0.001 and *s* to 1 (see above). We averaged the time until one of the two boundaries was reached and the number of boundary crossings over the 20,000 trials to obtain the mean RTs and response probabilities for correct and error responses.
